# Plant breeding at the speed of light: the power of CRISPR/Cas to generate directed genetic diversity at multiple sites

**DOI:** 10.1186/s12870-019-1775-1

**Published:** 2019-05-02

**Authors:** Felix Wolter, Patrick Schindele, Holger Puchta

**Affiliations:** Botanical Institute, Karlsruhe Institute of Technology, POB 6980, 76049 Karlsruhe, Germany

**Keywords:** Genome engineering, Plant breeding, CRISPR/Cas, Genetic diversity

## Abstract

Classical plant breeding was extremely successful in generating high yielding crop varieties. Yet, in modern crops, the long domestication process has impoverished the genetic diversity available for breeding. This is limiting further improvements of elite germplasm by classical approaches. The CRISPR/Cas system now enables promising new opportunities to create genetic diversity for breeding in an unprecedented way. Due to its multiplexing ability, multiple targets can be modified simultaneously in an efficient way, enabling immediate pyramiding of multiple beneficial traits into an elite background within one generation. By targeting regulatory elements, a selectable range of transcriptional alleles can be generated, enabling precise fine-tuning of desirable traits. In addition, by targeting homologues of so-called domestication genes within one generation, it is now possible to catapult neglected, semi-domesticated and wild plants quickly into the focus of mainstream agriculture. This further enables the use of the enormous genetic diversity present in wild species or uncultured varieties of crops as a source of allele-mining, widely expanding the crop germplasm pool.

## Background

For 10,000 years, humans have utilized the genetic diversity generated from spontaneous mutations and recombination for the selection of improved crops. These traditional breeding approaches have been extremely successful in delivering elite crop varieties with high yields and other enhanced traits, and even today, they remain the corner stone of plant breeding. In recent times, these classical breeding approaches could be accelerated by increasing selection efficiency using marker-assisted selection [1] and genomic selection [2]. However, the more knowledge we obtain about the underlying genomic factors of yield and quality, the more the limitations of these traditional breeding approaches become apparent. Due to the random nature of recombination and undirected mutagenesis, further improvement of current elite germplasm is a lengthy and tedious process. Introgression of beneficial traits into an elite variety is often impaired by linkage drag, the transfer of deleterious genetic material genetically linked to the desirable trait. This often necessitates multiple rounds of backcrossing and selection to restore the elite background, which is highly time- and cost intensive [3]. Furthermore, the efficiency of classical breeding approaches depends on the amount of available functional diversity, which is limited in many elite varieties that have passed through genetic bottlenecks during domestication [4]. Thus, the reliance on natural or randomly induced diversity is a limiting factor slowing down the breeding process [5] and contributing to an unpredictable breeding outcome [6]. In contrast, the highly precise nature of the genome editing technology CRISPR/Cas enables an unparalleled level of control over the mutation process, allowing immediate pyramiding of multiple beneficial traits into an elite background within one generation [7]. Additionally, direct improvement of elite varieties by genome editing does not introduce potentially deleterious alleles from crossing and recombination.

### The power of inducing site-specific DSBs

Already for classical breeding, the induction of DNA double-strand breaks (DSBs) by gamma irradiation was used to achieve genetic variability. The repair of these DSBs occurs in the large majority of cases by non-homologous end joining (NHEJ), which is error-prone [8]. It results in mutations such as deletions and insertions at the break site leading to new alleles that were not available before in the breeding population. Although most of these alleles were adverse for growth and/or yield, once and again mutations were isolated resulting in phenotypes that were attractive for breeders, such as cereals with shorter stems [9]. In the last two decades, classical transgenic approaches became available such as Agrobacterium-mediated transformation [10] or biolistic transformation [11, 12]. Thus, traits from utterly unrelated species became accessible. However, conventional mutation breeding and classical transgenic approaches are always non-specific as mutation and transgene insertion occur at random sites. Additionally, more modifications than the desired one are introduced. After it became clear that site-specific endonucleases can be used to induce DSBs in plant cells [13] resulting in directed mutagenesis of the plant genomes [14, 15], efforts were undertaken to target double strand breaks to specific genes of interest. This could be achieved by designing synthetic nucleases such as zinc-finger nucleases (ZFNs) and transcription activator-like effector nucleases (TALENs) [16]. However, the generation of genetic diversity on a large scale was only enabled through the characterization of the CRISPR/Cas-system. It makes use of the Cas9 nuclease that is guided by a programmable RNA to genomic sites of interest. Compared to the time-consuming and expensive cloning procedure of ZFNs and TALENs, the RNA based sequence specificity of the CRISPR/Cas-system enables cheap and fast adaptation to various sites and provides mutagenesis at high frequencies, also for plant genomes [17–21]. Potential disadvantages such as lower specificity can be compensated by customized systems such as paired nickases [22–24] or designed Cas9 variants [25, 26], highlighting the versatility of the system. As a consequence, numerous publications elucidated its potential for targeted mutagenesis and in particular for the improvement of qualitative traits in plants (for details see current reviews: [27–30]) For a comprehensive overview on crop traits modified by genome editing, see Zhang et al. [31]. Yet, the most outstanding feature represents its multiplexing applicability. Whereas ZFNs and TALENs are barely usable for multiplexing applications, the CRISPR/Cas9-system can be easily programmed to target several sites simultaneously [32–35]. This not only allows the manipulation of numerous traits in a single generation, but also provides access to the fine-tuning and optimization of relevant traits through targeted generation of genetic diversity.

### CRISPR enables immediate generation of genomic diversity for breeding

Several recent studies have demonstrated the potential of CRISPR/Cas to generate a broad range of allelic diversity at specific loci.

Shen et al. succeeded in editing eight yield or quality relevant genes in rice simultaneously [36]. Despite the high level of multiplexing, mutation rates in transgenic rice ranged from 50 to 100%. These high efficiencies allowed the isolation of mutants carrying homozygous mutated alleles of all eight targeted genes simultaneously. In addition to homozygous octuple mutants, septuple and sixtuple mutants as well as heterozygous mutants for all targeted genes were obtained. Thus, a wide range of different genotypes providing ample genetic diversity for selection could be generated within only one generation.

Another recent study showed that editing the same QTLs (Quantitative Trait Loci) can have different outcomes depending on the genetic background [37]. Two QTLs regulating grain size (*GRAIN SIZE3, GS3*) and grain number (Grain number 1a, Gn1a) were edited in five different widely cultivated rice varieties. Loss-of-function mutations in these QTLs were described to enhance yield [38, 39]. The authors report very high mutagenesis efficiency, which prevented the isolation of Gn1a single mutants, only allowing *GS3*/Gn1a double mutants and *GS3* single mutants to be isolated. Surprisingly, seven of the ten novel genotypes had decreased grain yield compared to the WT, indicating strong dependency of the editing outcome on genetic background and highlighting the utility of genetic diversity across different backgrounds.

Zhou et al. achieved simultaneous editing of three yield related QTLs in elite rice backgrounds [40]. They targeted the same two QTLs, *GS3* and Gn1a, in addition to *GRAIN WIDTH and WEIGHT 2* (*GW2*). All combinations of biallelic or homozygous single, double and triple mutants were obtained. The triple mutants showed increases in the yield related traits panicle length, flower number per panicle as well as grain length, width and weight. Unlike the study from Shen et al. [37], the resulting yield related phenotypic effects of the triple mutants were consistent across all 3 varieties employed in the study. This suggests that simultaneous disruption of these three genes could be used as a simple, generally applicable “formula” for yield increase in different varieties. However, for one of the three varieties the triple mutant showed a semi-dwarf phenotype, again suggesting background specific pleiotropic effects.

The multiplexing capability of CRISPR combined with its high efficiency in rice could recently be harnessed to create a system enabling the clonal reproduction from F1 hybrids, thus preserving the favourable high degree of heterozygosity [41]. Simultaneous targeting of three meiotic genes resulted in replacement of meiosis by a mitosis-like cell division generating clonal diploid gametes and tetraploid seeds. To prevent increase in ploidy, additional targeting of a gene involved in fertilization (*MATRILINEAL*), induced generation of clonal diploid seeds from hybrids that stably preserved heterozygosity.

As highlighted by another recent study, the polyploid nature of many crops can be a valuable source of genetic diversity [42]. The oil profile of the hexaploid oilseed crop *Camelina sativa* is dominated by polyunsaturated fatty acids and the development of new varieties rich in monounsaturated fatty acids is desirable. By targeting all three homeologs of the *CsFAD2* (*Fatty Acid Desaturase 2*) gene involved in fatty acid metabolism, a diverse set of genetic combinations with single, double and triple knockouts could be generated. The obtained lines varied strongly in their lipid profiles, with monounsaturated fatty acid levels in the oil ranging from 10%, like in wild type, up to 62% in homozygous triple mutants. As complete mutants with the strongest change in oil profile showed growth defects, the large mutant diversity could then be used for genetic fine-tuning of the trait, combining improved oil profile without growth defect.

### Creating new diversity in regulatory elements to generate a range of dosage effect alleles

**Fig. 1:**
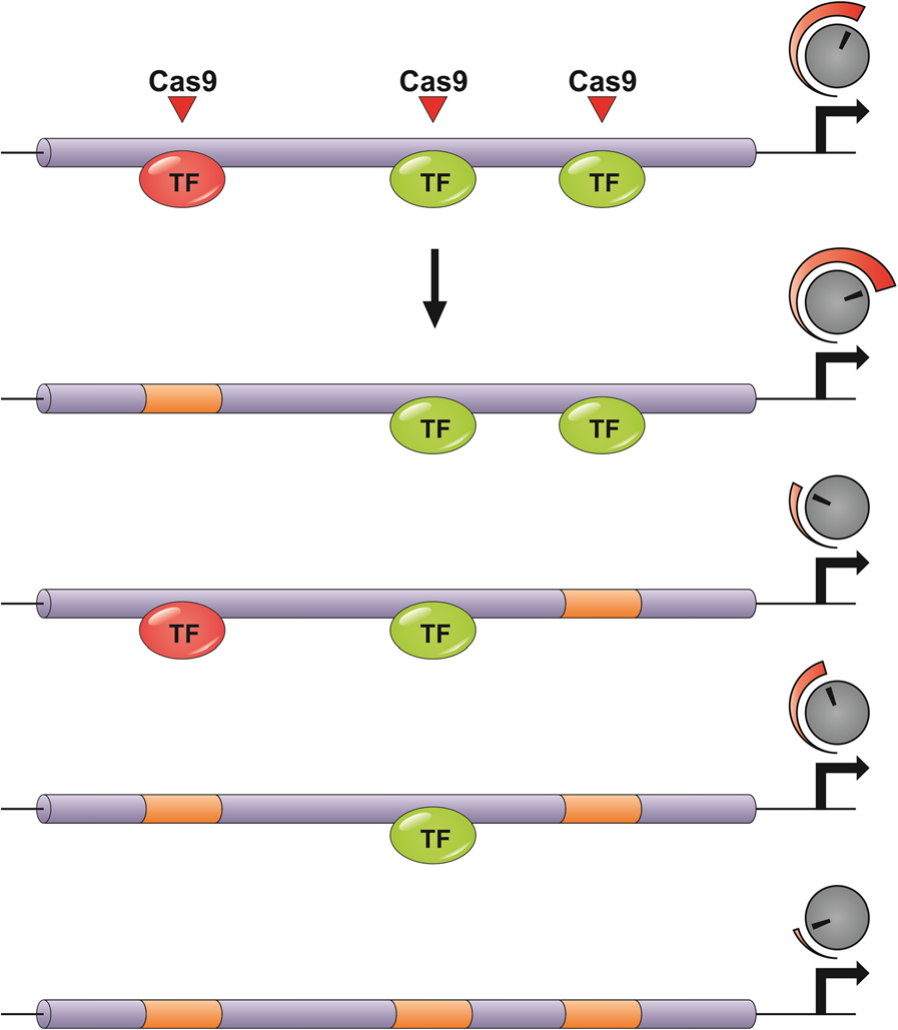
Editing of cis-regulatory elements for the generation of dosage effect alleles. In contrast to conventional editing of coding sequences, editing of cis-regulatory elements enables the fine-tuning towards optimal gene expression level. Red colour indicates repressive, green colour activating transcription factors. Red Triangles indicate CRISPR cleavage sites. Orange sections indicate CRISPR/Cas-induced mutations

Cis-regulatory elements are noncoding DNA sequences that contain binding sites for transcription factors or other molecules influencing transcription, the most common examples being promoters and enhancers. Promoters are generally bound by a common set of conserved transcription factors. In contrast, enhancers are much more variable. They can be located remote from the regulated gene and not only upstream but also downstream and even in introns [43]. Furthermore, enhancers are able to physically interact with target genes by altering chromatin state [44]. This regulatory part of the genome received much less attention than protein coding sequences in the past. However, several recent publications have demonstrated the enormous potential for crop improvement by editing regulatory sequences (see also [45]). Whereas classical knock-out mutations usually mediate complete loss-of-function with accompanying pleiotropic effects [46], editing regulatory elements offers the possibility to generate a range of alleles with varying expression intensity for precise fine-tuning of gene dosage (see Fig. 1).

In this regard, the Lippman lab at CSHL has recently achieved pioneering breakthroughs. First, they achieved optimization of inflorescence architecture in tomato by generating new weak transcriptional alleles [47]. They improved inflorescence architecture by combining two natural mutations mediating reduced expression of the tomato homologs of the Arabidopsis genes *SEPALLATA4* and *FRUITFULL*. The improved inflorescence architecture increased fruit number and weight as well as yield without a concomitant reduction in sugar content. Importantly, optimal inflorescence architecture could only be realized by a moderate increase in branching, which was dependent on alleles supporting reduced expression, one of them being in a heterozygous state. In contrast, combining CRISPR/Cas-mediated complete KO alleles in a homozygous state resulted in excessively branched inflorescences that produced infertile flowers. However, by targeting Cis-regulatory elements of above-mentioned genes with CRISPR, they generated a range of new alleles supporting varying expression levels for optimization of inflorescence architecture. The authors also identified a further promising Cis-regulatory element as editing target, *LIN*, which is another tomato *SEPALLATA4* homolog. Alleles conveying reduced *LIN* expression might enable subtle increases in flower production. The fact that rice carries a homolog of *LIN* that controls panicle architecture and grain production [48] suggests that the approach might be extended to other crop species.

Following this, the same group further developed this approach to a generally applicable genetic scheme for rapid generation and evaluation of novel transcriptional alleles [49]. In this system, a biallelic mutant is generated of the gene for which novel transcriptional alleles are desired. This mutant is transformed with a multiplex CRISPR system targeting the promoter of the gene of interest at many sites and crossed with WT. The progeny from the cross inherits one WT and one mutated allele that can be edited by Cas9. As the second allele is mutated, the transcriptional effect of novel mutations in the WT allele are immediately exposed in the phenotype. In the next generation, the transgene can be segregated out and novel transcriptional alleles can be fixed immediately, generating a population showing a wide variation of expression levels for the gene of interest in a transgene-free background. The broad feasibility and usefulness of this approach was demonstrated by applying the system to three genes regulating fruit size, inflorescence branching, and plant architecture. In all cases, a strong level of dosage sensitivity was observed. More strikingly, the relationship between gene dosage and phenotypic outcome was sometimes non-linear, indicating complex interactions in case of dose-sensitive developmental genes that function in complex regulatory networks [50], which further highlights the potential of targeting the promoters of other developmental regulators to modify diverse traits [49].

Fine-tuning of gene expression can also be achieved by targeting upstream ORFs (uORFs), short protein-coding elements located in the 5’UTR of an mRNA, upstream of the main ORF. Usually, uORFs act as post-transcriptional inhibitors of translation of the downstream pORF. They are quite widespread, in plants, around 30–40% of genes exhibit uORFs [51]. Now, the Gao lab demonstrated that CRISPR mediated disruption of uORFs can be used as a generally applicable means for increasing the production of a specific protein by enhancing translation of the respective mRNA [52]. In reporter gene assays, protein activity could be enhanced 8-fold by uORF disruption. The strategy also proved successful when it was applied to 4 different endogenous uORFs, two in Arabidopsis and two in lettuce. Agronomic relevance could also be shown by disruption of the uORF of LsGGP2, which encodes a key enzyme in vitamin C biosynthesis in lettuce. uORF disruption increased foliar ascorbic acid content by 157% and enhanced tolerance against oxidative stress.

### Opening up the genetic diversity from uncultured species

There are over 300,000 plant species. Less than 200 are used commercially, and only 3 species, wheat, rice and maize, provide most of the energy for human consumption [53, 54]. Further modification and improvement of elite varieties may not always be the most prudent path for generating new varieties adapted to altering conditions. In order to generate crops with novel properties, it could be highly useful to open up the enormous genetic diversity present in wild species or uncultured varieties from elite crop species by rapid domestication using genome editing. This applies especially to improvement of complex polygenic traits such as abiotic stress tolerance [55]. During the process of crop domestication, different crops have been selected for analogous traits such as favourable plant architecture and simultaneous flowering for simple harvest or large fruits for high yield. Our understanding of the genetic basis for these domestication traits is growing steadily and an increasing number of so-called domestication genes have been identified [54]. By targeting these genes with CRISPR, the domestication process can be accelerated dramatically. This is now finally possible, as demonstrated by three recent studies.

Zsögön et al. report de-novo domestication of the ancestral tomato relative *Solanum pimpinellifolium*, which exhibits a high degree of stress-tolerance [56]. Much of the genetic basis for stress tolerance was lost during the long domestication process of tomato. They used a multiplex CRISPR/Cas9 approach for simultaneous functional disruption of six domestication genes involved in plant architecture, yield components and nutritional quality. As in the other studies involving multiplex gene editing in tomato, efficiencies were extremely high since only mutated alleles were recovered. Compared to the wild parent, fruit size could be increased threefold and fruit number tenfold in a single generation and within a single transformation experiment. Furthermore, fruit shape was improved and nutritional quality enhanced by increasing lycopene content twofold, which translates to a fivefold increase compared to our modern cultivated tomato.

**Fig. 2:**
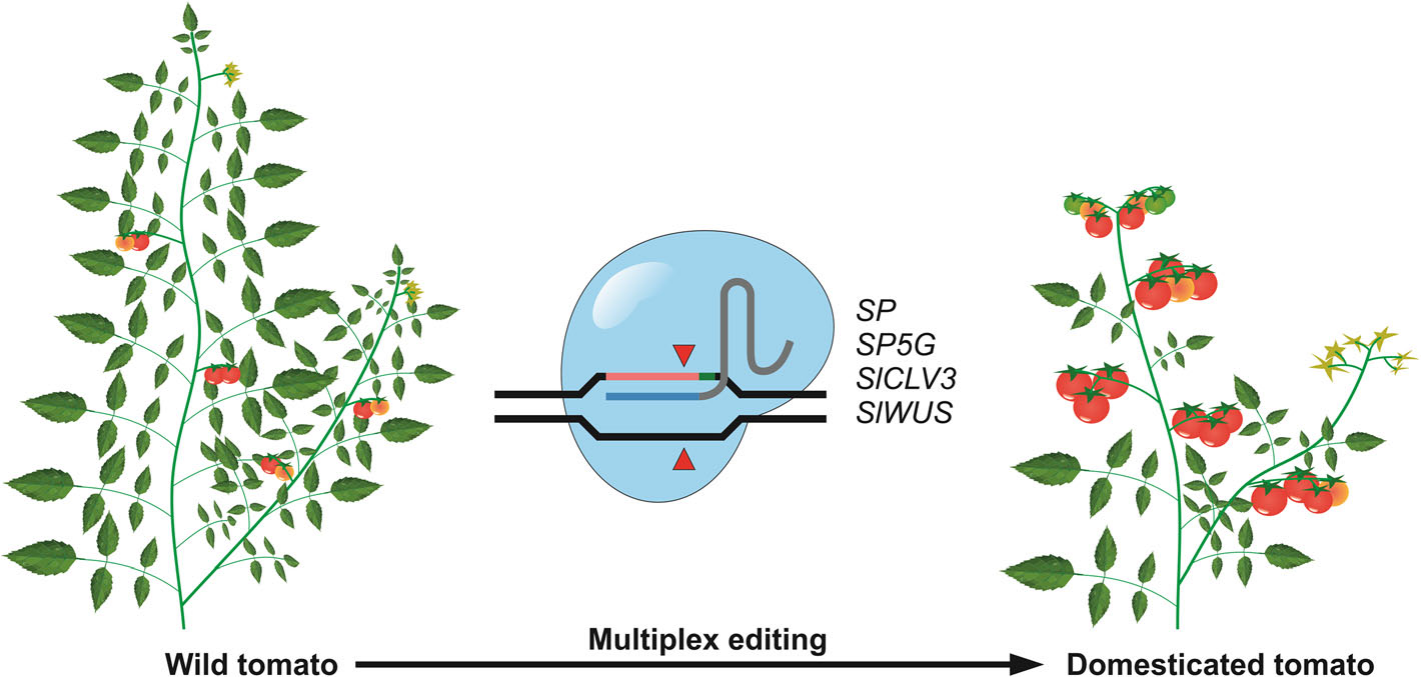
De-novo domestication of tomato by CRISPR/Cas9-mediated multiplex editing. By simultaneously editing four genes involved in plant architecture (SP), flowering time (SP5G) and fruit size (SlCLV3 and SlWUS), Li et al. [57] achieved accelerated domestication of wild tomato. Figure design according to Li et al. [57]

In the same issue of Nature Biotechnology, Li et al. report a similar approach for de-novo domestication of four wild tomato accessions each offering genetic diversity for resistance against specific stress conditions like bacterial spot disease or salt stress [57]. Using the multiplex capability of CRISPR, they simultaneously edited 4 target sites involved in plant architecture (*SP; SELF PRUNING*), flowering time (*SP5G; SELF PRUNING 5G*) and fruit size (*SlCLV3; CLAVATA3* and *SlWUS; WUSCHEL*), in all four accessions (see Fig. 2). In addition to targeting coding regions for loss-of-function mutations, they also targeted regulatory regions to generate weak transcriptional alleles. In the case of *SP* and *SP5G*, more than 100 mutated alleles were created allowing a continuum of flower production, fruit production and architecture to be generated within one generation. In contrast to Zsögön et al., who could only recover completely mutated plants due to high efficiency, Li et al. observed the whole range of combinations from only one mutated gene to all four genes mutated. The completely edited plants exhibited earlier and synchronized flowering, determinate growth architecture and increased fruit size, while retaining their original stress resistance.

More recently, rapid improvement of domestication traits hinting at de-novo domestication were undertaken in an orphan crop of the Solanaceae family, *Physalis pruinosa*, a striking achievement considering the previous lack of reference genome, gene annotation data and transformation protocol [58]. Initially, genomic resources had to be generated by whole genome sequencing and RNA sequencing de-novo assemblies, which subsequently enabled the identification of orthologues of domestication genes known from other Solanaceae crops. Three such genes were chosen as targets for genome editing, the *Physalis pruinosa* orthologues of *SP, SP5G* and *CLAVATA1* (*SlCLV1*). *SP* is a flowering repressor and weak alleles provide a compact determinate growth that enables simple mechanized harvesting. However, the effect from CRISPR generated null alleles *of Ppr-sp* was too strong, limiting fruit production similar to the null *sp* allele in tomato, where a weak transcriptional allele is optimal. *SP5G* was identified recently as an important domestication gene since null alleles eliminate day length sensitivity in tomato and other crops [59]. Concerning flowering, CRISPR *Ppr-sp5g* mutants did not show a useful effect. However, the mutants showed moderate shoot termination resulting in higher fruit amount along each shoot. The *Physalis* orthologue of *CLV1* was chosen as target for its involvement in the CLAVATA-WUSCHEL meristem size pathway influencing fruit size. Weak transcriptional *CLV3* alleles mediate enlarged fruits in many crops, whereas *clv3* null alleles mediate excessive and disorganized fruit production. Since *CLV1* acts as one of several redundant *CLV3* receptors, *clv1* null alleles might mimic weak transcriptional *CLV3* alleles. Indeed, the resulting *Ppr*-*clv1* mutants showed a 24% increase in fruit mass.

## Discussion

Opposed to traditional breeding approaches, improving crops by genome editing requires a much higher degree of genomic and bioinformatic knowledge, as it depends on functionally characterised candidate genes. But an increasing number of genes underlying QTLs is identified [60] and the more our knowledge about crop genomes grows, the more powerful CRISPR based breeding approaches become. In addition to genomic knowledge, improving crops by genome editing is dependent on efficient transformation and regeneration procedures. Accordingly, to harness the full potential of genome editing more effort is required to advance crop transformation [61]. The multiplex editing capability of CRISPR is an extremely valuable property, because it accelerates the breeding process enormously, and could be combined in this regard with double-haploid (DH) production [62] and speed breeding [5] to accelerate the process even further. DH lines are generated by crossing with an inducer line whose haploid chromosome set is lost in the zygote, followed by doubling the remaining haploid chromosome set, resulting in a completely homozygous plant being obtained in a single generation. One can imagine a breeding cycle consisting of multiplex genome editing followed by DH production for immediate homozygous fixation of the edited alleles, which might otherwise require multiple generations of selfing.

There are many traits that can be improved by simple knock-out mutations in the coding sequence of genes, but other traits require edits in regulatory sequences to generate new transcriptional alleles for fine-tuning of gene expression. To unlock the potential of changes in regulatory parts of the genome for dosage effects, the genetic scheme developed by the Lippman lab [49] seems highly promising. Traditionally, adapting desired allelic variants to diverse breeding germplasm is a cumbersome process. Now, with this novel genetic scheme the most desirable transcriptional allele can be generated directly and selected for in the context of the specific genetic background. Furthermore, it has much broader applicability beyond the generation of novel regulatory variants. The genetic scheme can be combined with any genome editing approach suitable for generating a set of novel variants at a specific location. In addition to the multiplex Cas9 approach used, it could be combined with paired or multiplex nickases, with the base editing system or the novel EvolvR system [63].

The base editing system enables precise C-to-T or A-to-G editing in a specified sequence range by fusion of Cas9 nickase with cytidine or adenine deaminase [64, 65]. Recently, the base-editing technology has been optimized further for plants by using human APOBEC3A as deaminase and additional minor modifications [66]. This enlarged the deamination window from protospacer position 3 to 9 to protospacer positions 1 to 17 and further enhanced the deamination efficiency in high GC sequence contexts. In addition, Zong et al. demonstrated the usefulness of base editing for generating new transcriptional alleles. Using their enhanced base editor, they targeted three regulatory elements in the *TaVRN1-A1* promoter in wheat protoplasts, which is involved in the regulation of vernalization. By deep-sequencing they identified a variety of mutations in all three targeted regulatory elements. Base-editing can also be used for elimination of specific splicing isoforms by inducing G to A conversions in the respective 5′ splice sites. This way, specific splicing events and the corresponding mature mRNA forms can be eliminated [67].

The EvolvR system provides another elegant way by which site-specific genetic diversity can be generated [63]. It relies on the fusion between an engineered error-prone polymerase domain to a Cas9 nickase. It enables the diversification of all nucleotides at a specific site and within a tuneable window length of up to 350 bp. In this window, the mutation rate can be elevated to more than 7 million times higher than in WT cells and using multiplexing, multiple loci can be diversified simultaneously. Accordingly, if only a large collection of random mutations is required at a specific locus, EvolvR has an advantage over base editing in terms of a larger diversity of mutations and a larger editing window.

Finally, CRISPR mediated de-novo domestication provides another new exciting possibility. On the one hand, this enables exploiting wild relatives of crops as a valuable source of allele mining, which could widely expand the crop germplasm pool. This should prove to be very useful considering the genetic impoverishment of many crops and the resistance of wild plants against a broad range of stresses [54]. On the other hand, de-novo domestication enables catapulting neglected, semi-domesticated, and wild plants into the focus of main-stream agriculture. Candidates for such an endeavour could be the grass teff, the pseudocereal amaranth or the legume cowpea [58]. A further candidate is pennycress, a common weed which could be converted into a cold-tolerant oilseed crop [68]. Additionally, progenitors of our elite crops such as teosinte (*Zea mays ssp. parviglumis*), wild emmer wheat (*Triticum dicoccoides*) and common wild rice (*Oryza rufipogon*) could be re-domesticated to generate novel varieties that retain lost traits. What is still limiting de-novo domestication efforts is the availability of efficient transformation procedures and genomic knowledge. However, the latter limitation will be overcome soon as an increasing number of wild species and minor crops are being sequenced. Finally, it should be noted that the trend of ever increasing homogeneization in modern agriculture might be suboptimal considering our changing climate [53]. Efforts of de-novo domestication and the concomitant general increase in crop diversity might soon prove to be the urgently needed antidote to the increasing crop uniformity.

Unfortunately, in many areas the development of new crop varieties by genome editing is hampered by strict GMO (Genetically Modified Organism) regulation, especially those areas adhering to a process rather than a product based regulatory framework, such as the European Union, where the authorization of new varieties developed by genome editing techniques is subjected to time- and cost intensive admission procedures. The recent ruling of the European Court of Justice decreed that targeted mutagenesis using genome editing tools is subject to the strict GMO legislation, even if the product is completely free of any transgene (ECJ 2018). This constitutes a considerable barrier to innovation and progress in these areas. In order to derive all the benefits from the new genome editing techniques and restore innovation, a switch to a product based regulatory framework is urgently needed in Europe. Fortunately, most other countries are not facing such an impediment to innovation, leaving no doubt that on a global scale CRISPR/Cas will continue to revolutionize plant breeding.

## Conclusion

The genetic bottlenecks imposed on our modern crops by the long domestication process have removed most of the genetic diversity available for breeding, which makes further improvement of elite varieties by traditional breeding technology a cumbersome process. CRISPR/Cas based new breeding tools including multiplex editing, fine-tuning of gene expression and de-novo domestication now provide plant breeders with exciting new opportunities to generate genetic diversity for breeding in an unprecedented way.

## Abbreviations

*CLV1*: *CLAVATA1*
*CLV3*: *CLAVATA3*
DSB: Double strand break
*FAD2*: *FATTY ACID DESATURASE 2*
GMO: Genetically modified organism
Gn1a: Grain number 1a
*GS3*: *GRAIN SIZE 3*
*GW2*: *GRAIN WIDTH AND WEIGHT 3*
NHEJ: Non homologous end joining
QTL: Quantitative trait locus
*SP*: *SELF PRUNING*
*SP5G*: *SELF PRUNING 5G*
TALEN: Transcription activator like effector nuclease
uORF: Upstream open reading frame
*WUS*: *WUSCHEL*
ZFN: Zinc finger nuclease

## References

[1] Collard BCY, Mackill DJ (2008). Marker-assisted selection: an approach for precision plant breeding in the twenty-first century. Philos Trans R Soc Lond Ser B Biol Sci.

[2] Desta ZA, Ortiz R (2014). Genomic selection: genome-wide prediction in plant improvement. Trends Plant Sci.

[3] Lidder P, Sonnino A (2012). Biotechnologies for the management of genetic resources for food and agriculture. Adv Genet.

[4] Shi J, Lai J (2015). Patterns of genomic changes with crop domestication and breeding. Curr Opin Plant Biol.

[5] Watson A, Ghosh S, Williams MJ, Cuddy WS, Simmonds J, Rey M-D (2018). Speed breeding is a powerful tool to accelerate crop research and breeding. Nat Plants.

[6] Scheben A, Edwards D (2018). Towards a more predictable plant breeding pipeline with CRISPR/Cas-induced allelic series to optimize quantitative and qualitative traits. Curr Opin Plant Biol.

[7] Zhang H, Zhang J, Wei P, Zhang B, Gou F, Feng Z (2014). The CRISPR/Cas9 system produces specific and homozygous targeted gene editing in rice in one generation. Plant Biotechnol J.

[8] Puchta H (2005). The repair of double-strand breaks in plants: mechanisms and consequences for genome evolution. J Exp Bot.

[9] Pacher M, Puchta H. From classical mutagenesis to nuclease-based breeding - directing natural DNA repair for a natural end-product. Plant J. 2016. 10.1111/tpj.13469. 10.1111/tpj.13469 28027431

[10] Herrera-Estrella L, Depicker A, van Montagu M, Schell J (1983). Expression of chimaeric genes transferred into plant cells using a Ti-plasmid-derived vector. Nature.

[11] Klein TM, Fromm M, Weissinger A, Tomes D, Schaaf S, Sletten M, Sanford JC (1988). Transfer of foreign genes into intact maize cells with high-velocity microprojectiles. Proc Natl Acad Sci U S A.

[12] Klein TM, Harper EC, Svab Z, Sanford JC, Fromm ME, Maliga P (1988). Stable genetic transformation of intact Nicotiana cells by the particle bombardment process. Proc Natl Acad Sci U S A.

[13] Puchta H, Dujon B, Hohn B (1993). Homologous recombination in plant cells is enhanced by in vivo induction of double strand breaks into DNA by a site-specific endonuclease. Nucl Acids Res.

[14] Salomon S, Puchta H (1998). Capture of genomic and T-DNA sequences during double-strand break repair in somatic plant cells. EMBO J.

[15] Puchta H, Dujon B, Hohn B (1996). Two different but related mechanisms are used in plants for the repair of genomic double-strand breaks by homologous recombination. Proc Natl Acad Sci U S A.

[16] Voytas DF (2013). Plant genome engineering with sequence-specific nucleases. Annu Rev Plant Biol.

[17] Jinek M, Chylinski K, Fonfara I, Hauer M, Doudna JA, Charpentier E (2012). A programmable dual-RNA-guided DNA endonuclease in adaptive bacterial immunity. Science.

[18] Li J-F, Norville JE, Aach J, McCormack M, Zhang D, Bush J (2013). Multiplex and homologous recombination-mediated genome editing in Arabidopsis and Nicotiana benthamiana using guide RNA and Cas9. Nat Biotechnol.

[19] Nekrasov V, Staskawicz B, Weigel D, Jones JDG, Kamoun S (2013). Targeted mutagenesis in the model plant Nicotiana benthamiana using Cas9 RNA-guided endonuclease. Nat Biotechnol.

[20] Shan Q, Wang Y, Li J, Zhang Y, Chen K, Liang Z (2013). Targeted genome modification of crop plants using a CRISPR-Cas system. Nat Biotechnol.

[21] Fauser F, Schiml S, Puchta H (2014). Both CRISPR/Cas-based nucleases and nickases can be used efficiently for genome engineering in Arabidopsis thaliana. Plant J.

[22] Mali P, Aach J, Stranges PB, Esvelt KM, Moosburner M, Kosuri S (2013). CAS9 transcriptional activators for target specificity screening and paired nickases for cooperative genome engineering. Nat Biotechnol.

[23] Ran FA, Hsu PD, Lin C-Y, Gootenberg JS, Konermann S, Trevino AE (2013). Double nicking by RNA-guided CRISPR Cas9 for enhanced genome editing specificity. Cell.

[24] Schiml S, Fauser F, Puchta H (2014). The CRISPR/Cas system can be used as nuclease for in planta gene targeting and as paired nickases for directed mutagenesis in Arabidopsis resulting in heritable progeny. Plant J.

[25] Slaymaker IM, Gao L, Zetsche B, Scott DA, Yan WX, Zhang F (2016). Rationally engineered Cas9 nucleases with improved specificity. Science.

[26] Kleinstiver BP, Pattanayak V, Prew MS, Tsai SQ, Nguyen NT, Zheng Z, Joung JK (2016). High-fidelity CRISPR-Cas9 nucleases with no detectable genome-wide off-target effects. Nature.

[27] Schindele P, Wolter F, Puchta H (2018). Transforming plant biology and breeding with CRISPR/Cas9, Cas12 and Cas13. FEBS Lett.

[28] Kumlehn J, Pietralla J, Hensel G, Pacher M, Puchta H (2018). The CRISPR/Cas revolution continues: from efficient gene editing for crop breeding to plant synthetic biology. J Integr Plant Biol.

[29] Yin K, Gao C, Qiu J-L (2017). Progress and prospects in plant genome editing. Nat Plants.

[30] Langner T, Kamoun S, Belhaj K (2018). CRISPR crops: plant genome editing toward disease resistance. Annu Rev Phytopathol.

[31] Zhang Y, Massel K, Godwin ID, Gao C (2018). Applications and potential of genome editing in crop improvement. Genome Biol.

[32] Li J, Stoddard TJ, Demorest ZL, Lavoie P-O, Luo S, Clasen BM (2016). Multiplexed, targeted gene editing in Nicotiana benthamiana for glyco-engineering and monoclonal antibody production. Plant Biotechnol J.

[33] Bao Z, Cobb RE, Zhao H (2016). Accelerated genome engineering through multiplexing. Wiley Interdiscip Rev Syst Biol Med.

[34] Soyars CL, Peterson BA, Burr CA, Nimchuk ZL (2018). Cutting edge genetics: CRISPR/Cas9 editing of plant genomes. Plant Cell Physiol.

[35] Zhang Z, Mao Y, Ha S, Liu W, Botella JR, Zhu J-K (2016). A multiplex CRISPR/Cas9 platform for fast and efficient editing of multiple genes in Arabidopsis. Plant Cell Rep.

[36] Shen L, Hua Y, Fu Y, Li J, Liu Q, Jiao X (2017). Rapid generation of genetic diversity by multiplex CRISPR/Cas9 genome editing in rice. Sci China Life Sci.

[37] Shen L, Wang C, Fu Y, Wang J, Liu Q, Zhang X (2018). QTL editing confers opposing yield performance in different rice varieties. J Integr Plant Biol.

[38] Takano-Kai N, Jiang H, Kubo T, Sweeney M, Matsumoto T, Kanamori H (2009). Evolutionary history of GS3, a gene conferring grain length in rice. Genetics.

[39] Ashikari M, Sakakibara H, Lin S, Yamamoto T, Takashi T, Nishimura A (2005). Cytokinin oxidase regulates rice grain production. Science.

[40] Zhou J, Xin X, He Y, Chen H, Li Q, Tang X, et al. Multiplex QTL editing of grain-related genes improves yield in elite rice varieties. Plant Cell Rep. 2018. 10.1007/s00299-018-2340-3. 10.1007/s00299-018-2340-3 30159598

[41] Wang C, Liu Q, Shen Y, Hua Y, Wang J, Lin J, et al. Clonal seeds from hybrid rice by simultaneous genome engineering of meiosis and fertilization genes. Nat Biotechnol. 2019;1. 10.1038/s41587-018-0003-0. 10.1038/s41587-018-0003-0 30610223

[42] Morineau C, Bellec Y, Tellier F, Gissot L, Kelemen Z, Nogué F, Faure J-D (2017). Selective gene dosage by CRISPR-Cas9 genome editing in hexaploid Camelina sativa. Plant Biotechnol J.

[43] Wittkopp PJ, Kalay G (2012). *Cis*-regulatory elements: molecular mechanisms and evolutionary processes underlying divergence. Nat Rev Genet.

[44] Fulco CP, Munschauer M, Anyoha R, Munson G, Grossman SR, Perez EM (2016). Systematic mapping of functional enhancer-promoter connections with CRISPR interference. Science.

[45] Wolter F, Puchta H (2018). Application of CRISPR/Cas to understand Cis- and trans-regulatory elements in plants. Methods Mol Biol.

[46] Meyer RS, Purugganan MD (2013). Evolution of crop species: genetics of domestication and diversification. Nat Rev Genet.

[47] Soyk S, Lemmon ZH, Oved M, Fisher J, Liberatore KL, Park SJ (2017). Bypassing negative epistasis on yield in tomato imposed by a domestication gene. Cell.

[48] Liu C, Teo ZWN, Bi Y, Song S, Xi W, Yang X (2013). A conserved genetic pathway determines inflorescence architecture in Arabidopsis and rice. Dev Cell.

[49] Rodríguez-Leal D, Lemmon ZH, Man J, Bartlett ME, Lippman ZB (2017). Engineering quantitative trait variation for crop improvement by genome editing. Cell.

[50] Birchler JA, Johnson AF, Veitia RA (2016). Kinetics genetics: incorporating the concept of genomic balance into an understanding of quantitative traits. Plant Sci.

[51] von Arnim AG, Jia Q, Vaughn JN (2014). Regulation of plant translation by upstream open reading frames. Plant Sci.

[52] Zhang H, Si X, Ji X, Fan R, Liu J, Chen K (2018). Genome editing of upstream open reading frames enables translational control in plants. Nat Biotechnol.

[53] Massawe F, Mayes S, Cheng A (2016). Crop diversity: an unexploited treasure trove for food security. Trends Plant Sci.

[54] Østerberg JT, Xiang W, Olsen LI, Edenbrandt AK, Vedel SE, Christiansen A (2017). Accelerating the domestication of new crops: feasibility and approaches. Trends Plant Sci.

[55] Scheben A, Wolter F, Batley J, Puchta H, Edwards D (2017). Towards CRISPR/Cas crops - bringing together genomics and genome editing. New Phytol.

[56] Zsögön A, Čermák T, Naves ER, Notini MM, Edel KH, Weinl S, et al. *De novo* domestication of wild tomato using genome editing. Nat Biotechnol. 2018. 10.1038/nbt.4272. 10.1038/nbt.4272 30272678

[57] Li T, Yang X, Yu Y, Si X, Zhai X, Zhang H, et al. Domestication of wild tomato is accelerated by genome editing. Nat Biotechnol. 2018. 10.1038/nbt.4273. 10.1038/nbt.4273 30272676

[58] Lemmon ZH, Reem NT, Dalrymple J, Soyk S, Swartwood KE, Rodriguez-Leal D (2018). Rapid improvement of domestication traits in an orphan crop by genome editing. Nat Plants.

[59] Soyk S, Muller NA, Park SJ, Schmalenbach I, Jiang K, Hayama R (2017). Variation in the flowering gene SELF PRUNING 5G promotes day-neutrality and early yield in tomato. Nat Genet.

[60] Nogué F, Mara K, Collonnier C, Casacuberta JM (2016). Genome engineering and plant breeding: impact on trait discovery and development. Plant Cell Rep.

[61] Altpeter F, Springer NM, Bartley LE, Blechl AE, Brutnell TP, Citovsky V (2016). Advancing crop transformation in the era of genome editing. Plant Cell.

[62] Dwivedi SL, Britt AB, Tripathi L, Sharma S, Upadhyaya HD, Ortiz R (2015). Haploids: constraints and opportunities in plant breeding. Biotechnol Adv.

[63] Halperin SO, Tou CJ, Wong EB, Modavi C, Schaffer DV, Dueber JE (2018). CRISPR-guided DNA polymerases enable diversification of all nucleotides in a tunable window. Nature.

[64] Komor AC, Kim YB, Packer MS, Zuris JA, Liu DR (2016). Programmable editing of a target base in genomic DNA without double-stranded DNA cleavage. Nature.

[65] Gaudelli NM, Komor AC, Rees HA, Packer MS, Badran AH, Bryson DI, Liu DR (2017). Programmable base editing of A•T to G•C in genomic DNA without DNA cleavage. Nature.

[66] Zong Y, Song Q, Li C, Jin S, Zhang D, Wang Y (2018). Efficient C-to-T base editing in plants using a fusion of nCas9 and human APOBEC3A. Nat Biotechnol.

[67] Xue C, Zhang H, Lin Q, Fan R, Gao C (2018). Manipulating mRNA splicing by base editing in plants. Sci China Life Sci.

[68] Sedbrook JC, Phippen WB, Marks MD (2014). New approaches to facilitate rapid domestication of a wild plant to an oilseed crop: example pennycress (Thlaspi arvense L.). Plant Sci.

